# Der querschnittgelähmte Patient – Besonderheiten der viszeralchirurgischen Diagnostik und Therapie

**DOI:** 10.1007/s00104-021-01364-2

**Published:** 2021-02-25

**Authors:** Julia Seifert, Ralf Böthig, Stefan Wolter, Jakob R. Izbicki, Roland Thietje, Michael Tachezy

**Affiliations:** 1 Abteilung für Allgemein und Viszeralchirurgie, BG Klinikum Hamburg, Hamburg, Deutschland; 2 Klinik für Allgemein‑, Viszeral- und Thoraxchirurgie, Universitätsklinikum Hamburg-Eppendorf, Martinistraße 52, 20246 Hamburg, Deutschland; 3 Abteilung für Neuro-Urologie des Querschnittgelähmten-Zentrum, BG Klinikum Hamburg, Hamburg, Deutschland; 4 Querschnittgelähmten-Zentrum, BG Klinikum Hamburg, Hamburg, Deutschland

**Keywords:** Neurogene Darmfunktionsstörung, Spinaler Schock, Obstipation, Diagnostik, Läsion oberes und unteres Motoneuron, Neurogenic bowel dysfunction, Spinal shock, Constipation, Diagnostics, Upper and lower motor neuron lesion

## Abstract

**Hintergrund:**

Patienten mit einer Querschnittlähmung entwickeln syndromspezifische, viszeralchirurgisch relevante Krankheitsbilder, die im Rahmen des akuten spinalen Schocks auftreten können oder Folge der zumeist progredienten neurogenen Darmfunktionsstörung (NBD) mit Ausbildung eines Colon elongatum und/oder Megakolons sind. Auch die akuten abdominalchirurgischen Notfälle, wie akute Appendizitis, Cholezystitis, Divertikultis oder Ileusbilder, stellen den Kliniker bei untypischer oder teils fehlender Klinik vor diagnostische und therapeutische Herausforderungen. Einen zunehmenden Stellenwert nimmt die Ausweitung adipositaschirurgischer Indikationen auf Patienten mit einer Querschnittlähmung ein

**Ziel der Arbeit:**

Dieser Artikel soll einen Überblick über die speziellen Anforderungen und Aspekte in der Behandlung dieses speziellen Patientenkollektivs geben und die Evidenz querschnittspezifischer viszeralchirurgischer Behandlungen aufzeigen.

**Material und Methoden:**

Es wurde eine selektive Literaturrecherche in den Datenbanken Medline und Cochrane Library in deutscher und englischer Sprache (1985–2020) durchgeführt.

**Ergebnisse und Diskussion:**

Die klinische Behandlung querschnittgelähmter Patienten erfordert einen profunden Kenntnisstand über die pathophysiologischen Veränderungen bei unterschiedlicher Querschnitthöhe (oberes vs. unteres motorisches Neuron) und die Phasen der Erkrankung (spinaler Schock vs. Langzeitverlauf). Fehlende oder atypische klinische Symptome akuter Erkrankungen verzögern eine rasche Diagnosefindung und machen eine frühzeitige Durchführung gezielter Diagnostik unabdingbar. Die Evidenz der chirurgischen Behandlung der akuten und chronischen Folgen der NBD ist gering und basiert auf Fallserien und „case reports“ ebenso wie die für spezielle Indikationen wie adipositaschirurgische Eingriffe.

## Abstract (en)

**Background:**

Patients with paraplegia develop syndrome-specific complications relevant to visceral surgery, which occur in the context of the acute spinal shock or as a consequence of the progressive neurogenic bowel dysfunction (NBD) with the formation of an elongated colon and/or megacolon. Moreover, acute abdominal emergencies, such as acute appendicitis, cholecystitis, diverticulitis and ileus images, pose particular challenges for the clinician when the clinical signs are atypical or even absent. The expansion of indications for obesity surgery to include patients with a paraplegic syndrome, whose independence and quality of life can be impaired due to the restricted mobility, especially by obesity, is becoming increasingly more important.

**Objective:**

This article provides an overview of the special requirements and aspects in the treatment of this special patient collective and to show the evidence of paraplegia-specific visceral surgery treatment.

**Material and methods:**

Targeted literature search in Medline and Cochrane library (German and English, 1985–2020).

**Results and conclusion:**

The clinical treatment of paraplegic patients requires in-depth knowledge of the pathophysiological changes at the different height of the paraplegia (upper versus lower motor neuron) and the phase of the disease (spinal shock versus long-term course). Missing or atypical clinical symptoms of acute diseases delay a quick diagnosis and make early diagnosis essential. The evidence for surgical treatment of the acute and chronic consequences of NBD is based on small retrospective series and case reports, as is that for special indications such as obesity surgery.

## Hintergrund

Pro Jahr kommt es in Deutschland zu etwa 2400 Querschnittlähmungen, wobei die Lebenserwartung nach dem ersten Jahr mit deutlich erhöhter Mortalität mit der Normalpopulation vergleichbar ist [18, 21]. Die inzwischen etwa 140.000 in Deutschland lebenden Menschen mit Querschnittsyndrom machen einen relevanten Anteil der Bevölkerung aus und so nimmt die Behandlung auch außerhalb spezialisierter Zentren zu [18]. Erfreulicherweise wird dem Thema in den letzten Jahren durch Politik, Presse und unabhängige Stiftungen eine erhöhte Aufmerksamkeit geschenkt.

Die viszeralchirurgische Beurteilung und Behandlung von Menschen mit einer Querschnittlähmung unterscheidet sich nicht unerheblich von Patienten ohne Lähmungssyndrom, nicht nur infolge der eingeschränkten Untersuchungsmöglichkeiten, sondern eben auch aufgrund der drastisch veränderten neurologischen Regulationsmechanismen. Wir wollen mit diesem Artikel auf die Besonderheiten dieses speziellen Patientenkollektivs eingehen und auch damit verbundene Berührungsängste und Unsicherheiten abbauen, die diese Patienten nicht selten in einem damit ungeübten klinischen Umfeld erfahren.

Aus viszeralchirurgischer Sicht stellt vor allem die Diagnostik und Therapie der akuten und chronische Veränderungen der neurogenen Darmfunktionsstörung („neurogenic bowel dysfunction“, NBD) eine Besonderheit dar, aber auch die Eigenheiten in der Therapie und Diagnostik des akuten Abdomens. Zuletzt stellen wir die Evidenz bariatrischer Operationen bei Patienten mit Querschnittlähmung vor, die bei den ohnehin in ihrer Mobilität eingeschränkten Patienten deutliche Verbesserungen der Lebensqualität erbringen können.

## Literaturrecherche

Wir führten eine selektive Literaturrecherche mit Schwerpunkt auf die viszeralchirurgische Therapie von Menschen mit Querschnittlähmung im Zeitraum 1985 bis Ende 2020 in Medline durch (MeSH-Terms: „spinal cord injury“, „paraplegia“, „neurogenic bowel dysfunction“, „acute abdomen“, „surgery“, „constipation“). Es finden sich neben Übersichtsarbeiten zu einzelnen Themen lediglich retrospektive Fallserien und „case reports“.

## Ergebnisse

### Pathophysiologie und Einteilung der Querschnittsyndrome

Alle Erkrankungen oder Verletzungen, die eine Schädigung des Rückenmarks bewirken, können ein reversibles oder irreversibles Querschnittsyndrom hervorrufen.

Dem kompletten Querschnittsyndrom liegt die völlige Unterbrechung der Leitfähigkeit des Rückenmarks zugrunde mit der Folge des Verlustes der neurogenen Steuerung der distal der Läsion gelegenen Körperfunktionen.

Die Querschnittsyndrome können auf verschiedene Weise klassifiziert werden. Zum einen wird in komplette oder inkomplette Querschnittlähmungen unterteilt. Zum anderen kann nach Art der Verletzung oder Ätiologie unterschieden werden, in ca. der Hälfte der Fälle liegt eine traumatische Schädigung vor [22]. Andere Ursachen können Tumoren, Entzündungen, gefäßbedingte oder degenerative Erkrankungen sowie kongenitale Fehlbildungen oder auch iatrogene Schädigungen sein.

Am geläufigsten ist die Klassifikation und Verlaufsbeschreibung der Querschnittsymptomatik anhand der Lähmungshöhe und des ASIA-Schemas (American Spinal Injury Association;[29]; Tab. 1).

**Table 1:** 

Einteilung	Erklärung
A	Komplett: Keine sensible oder motorische Funktion ist in den sakralen Segmenten S4–S5 erhalten
B	Inkomplett: Sensible, aber keine motorische Funktion unterhalb des neurologischen Niveaus erhalten; dehnt sich bis in die sakralen Segmente S4–S5 aus
C	Inkomplett: Motorische Funktion ist unterhalb des neurologischen Niveaus erhalten und die Mehrzahl der Kennmuskeln unterhalb des neurologischen Niveaus haben einen Muskelkraftgrad < 3
D	Inkomplett: Motorische Funktion ist unterhalb des Schädigungsniveaus erhalten und die Mehrheit der Kennmuskeln unterhalb des neurologischen Niveaus haben einen Muskelkraftgrad ≥ 3
E	Normal: Sensible und motorische Funktion ist normal

*ASIA *American Spinal Injury Association

Es existiert eine weitere Klassifizierung für komplett Querschnittgelähmte, welche stärker auf neuropathophysiologischen Gesichtspunkten mit auch viszeralchirurgischer Relevanz beruht ([54]; Tab. 2).

**Table 2:** 

Lähmungshöhe	Details
> Th7	Keine Bauchpresse, häufig autonome Dysreflexie
< Th7	Sakrale Reflexe erhalten
< Th7	Sakrale Reflexe erloschen

Prinzipiell kann man zwei Phasen unterscheiden:

- die akute Querschnittlähmung mit spinalem Schockgeschehen und
- die chronischen Langzeitfolgen mit ihren unterschiedlichen Auswirkungen auf die Darmfunktion.

### Spinaler Schock

Die Dauer des spinalen Schocks variiert von Stunden bis zu Monaten, durchschnittlich handelt es sich um 4 bis 6 Wochen [15]. Es kommt zunächst zur schlaffen Lähmung der Muskulatur, dem Fehlen von Fremd- und Eigenreflexen und einer gestörten Kreislauf- und Thermoregulation.

In der Phase des spinalen Schocks kommt es zur Darmatonie, häufig bis zur kompletten Paralyse. Die zum Zeitpunkt des spinalen Schocks im Kolon vorhandene Stuhlsäule kann nicht weiter transportiert werden. Somit wird sie dann von der regulären Darmflora erneut verarbeitet mit der Folge einer Gärung und dem Auftreten eines massiven Meteorismus.

Die konservative Behandlung des frisch gelähmten Darmes erfordert viel Geduld und Erfahrung. Die Indikation chirurgischer Maßnahmen sollte auf Organkomplikationen der Paralyse wie Ischämie, Durchwanderungsperitonitis oder Perforation begrenzt sein, zu denen es in 2–11 % aller Fälle kommt, zumeist früh nach Eintreten der Schädigung [35]. Diese werden in etwa 10 % zu spät oder gar nicht erkannt [47]. Risikofaktoren hierfür sind hohes Alter, männliches Geschlecht, zervikale Verletzungen und Polytraumata [35].

### Autonome Dysreflexie

Eine Besonderheit stellt das Auftreten der autonomen Dysreflexie, zumeist bei Patienten mit einer Querschnittlähmung > Th6, dar, die durch Schmerzreize oder andere starke Reizzustände unterhalb der Läsion (Blasenprobleme, Darmdehnung, abdominelle Entzündungen, Hämorrhoiden u. a.) ausgelöst werden kann [14, 26]. Durch eine exzessive Ausschüttung von Stresshormonen kommt es zu einer potenziell lebensbedrohlichen hypertensiven Krise (oft mit Bradykardie), die sich zumeist mit Kopfschmerzen und vermehrtem Schwitzen ankündigt. Neben der Beseitigung oder Meidung der auslösenden Faktoren muss eine rasche Blutdrucksenkung erfolgen.

### Neurogene Darmfunktionsstörung

Bei einer Mehrzahl aller Querschnittgelähmten treten gastrointestinale Probleme (Obstipation, Inkontinenz, Meteorismus, Übelkeit, Diarrhö, Bauchschmerzen, rektale Blutabgänge, Hämorrhoiden) auf. Die Veränderungen der gastrointestinalen Funktionen sind in ihrer klinischen Erscheinung abhängig von der Höhe der Läsion, dem Verletzungsausmaß und dem Zeitraum, der seit der Querschnittlähmung vergangen ist. Klinisch entscheidend ist, ob Motilität und Peristaltik des Gastrointestinaltrakts erhalten sind, sowie die Sphinkterfunktion.

Zum Verständnis der unterschiedlichen Ausprägungen der NBD müssen die Läsionen des oberen und unteren motorischen Neurons in ihrer Symptomatik und Therapie gegenübergestellt werden [15]. Läsionen des oberen Motoneurons liegen oberhalb des sakralen Rückenmarkes und führen zum sog. „reflexiven Darm“. Das sakrale Reflexzentrum ist intakt. Daraus resultiert ein erhöhter Tonus von Beckenboden und Sphinkter. Die reflektorische Peristaltik des Darms ist vorhanden. Anal- und Bulbokavernosusreflex bleiben erhalten. Die Defäkation ist mittels rektaler Stimulation anregbar. Die Patienten leiden aufgrund des erhöhten Tonus der Beckenboden- und Sphinktermuskulatur eher an einer „outlet constipation“. Wobei es hier auch zu einer Überlaufinkontinenz kommen kann [54].

Demgegenüber stehen die Läsionen des unteren Motoneurons, der sog. „areflexive Darm“. Hier kommt es zur Zerstörung der Nerven im Conus medullaris oder der Nervenfasern der Cauda equina. In dieser Situation gibt es keine reflektorische Peristaltik und somit auch keine Reflexentleerung. Nur noch der Plexus myentericus kann zum langsamen Transport des Stuhls beitragen. Beckenboden und Sphinkter sind denerviert. Somit leiden die Patienten an einer neurogenen Inkontinenz („passive leakage“). Der deutlich verlangsamte Stuhltransport stellt sich als „slow transit constipation“ dar.

### Diagnostik der neurogenen Darmfunktionsstörung

Essenziell wichtig für die Therapie der NBD ist also zu wissen, in welcher Höhe die Schädigung des Rückenmarks liegt. Zur Einordnung der Beschwerden gehört eine spezifische Anamneseerhebung in Bezug auf die aktuellen Beschwerden, die Stuhlfrequenz und -beschaffenheit (Bristol-Skala). Neben dem sog. NBD-Score wird in der S2k-Leitlinie der ausführlichere, aber bisher nicht validierte nDFS(neurogene Darmfunktionsstörung)-Selbsterhebungsbogen empfohlen [15, 30]. Des Weiteren sollten Stuhl‑, Ernährungs- und Trinkprotokolle angelegt werden.

Die klinische Untersuchung sollte eine digital rektale Untersuchung zur Überprüfung der perianalen sowie der tiefen Sensibilität, des Sphinkterruhetonus und der willentlichen Kontraktilität sowie die Prüfung des Bulbokavernosus- und Analreflexes einschließen. Diese Untersuchung beinhaltet auch die Beurteilung häufiger Komorbiditäten wie Hautveränderungen, Blutungen, Fissuren, Hämorrhoidalleiden, Anal- und Rektumprolaps. Die Behandlung dieser lokalen Komplikationen unterscheidet sich jedoch nicht von der nicht gelähmter Patienten.

Zur erweiterten Diagnostik der neurogenen Darmfunktionsstörung gehören Abdomensonographie und -röntgen, Laborparameter, Proktorektoskopie bzw. Koloskopie und die Computertomographie (CT) des Abdomens. Bei speziellen Fragestellungen kann auf Rektomanometrie, Kolontransitzeit, Kolonkontrasteinlauf, Magnetresonanz(MR)-Defäkographie, Endosonographie oder Elektromyographie zurückgegriffen werden.

### Therapie der neurogenen Darmfunktionsstörung

Die Therapie der NBD folgt leitlinienkonform einem Stufenschema und sollte erst nach Abklingen des spinalen Schocks in dieser Form durchgeführt werden [15]. Die drei vorrangigen Ziele sind

1. regelmäßige, planbare, zeitlich begrenzte Darmentleerungen mit adäquater Stuhlmenge und -konsistenz,
2. die Vermeidung einer Stuhlinkontinenz, sodass die
3. Lebensqualität so gering wie möglich beeinträchtigt wird [7].

Zu Beginn dieses multiprofessionellen Darmmanagements, das bei 95 % aller Betroffenen erforderlich ist, stehen konservative Maßnahmen [19]: Die ersten Schritte sind die Optimierung einer ballaststoffreichen Ernährung und des Flüssigkeitshaushalts, Lifestyle-Veränderungen (körperliche Aktivität), physiotherapeutische Maßnahmen sowie das Ausnutzen des gastrokolischen Refexes. Des Weiteren können Kolonmassagen, eine medikamentöse Stuhlregulation (Macrogol, Lactulose) oder auch ein digitales Ausräumen hilfreich sein.

Nur bei Läsionen des oberen Motoneurons kommen CO_2_-Suppositorien (Dehnungsreiz!), digitale Stimulation, Mikroklistiere oder Klysmata infrage. Die nächsthöhere Stufe für beide Läsionen ist die transanale Irrigation, die allerdings Auslöser einer autonomen Dysreflexie sein kann (s. oben; [14, 26]).

Für eine vertiefende Betrachtung der konservativen Therapiemaßnahmen des Darmmanagements sei auf die S2k-Leitlinie der Deutschsprachigen Medizinischen Gesellschaft für Paraplegiologie verwiesen [15].

Die NBD führt nicht selten und mit fortschreitender Dauer der Querschnittlähmung zu einer zunehmenden Einschränkung der Lebensqualität einschließlich sozialer Teilhabe und Unabhängigkeit, da die konservativen Abführregime keine zuverlässigen Stuhlentleerungen in einer akzeptablen Zeit mehr erlauben und es vermehrt zu einer Stuhlinkontinenz (in etwa 75 %) kommt [12, 31]. Bei entsprechendem Leidensdruck der Patienten mit ihrer individuellen Symptomatik, kommen interventionelle und operative Maßnahmen zum Einsatz.

#### Botox-Injektionen und Sakralnervenstimulation

Die Botox-Injektion bei spastischem Analsphinkter kommt nur für Patienten mit Läsionen des oberen motorischen Neurons infrage [15]. Bei Patienten mit inkompletter Lähmung könnte die Sakralnervenmodulation (SNM) dazu beitragen, neben der besser validierten Wirkung auf die Blasen- auch die Darmfunktion zu verbessern, wobei sich die Evidenz aus kleinen Fallserien begründet und dementsprechend keine klare Empfehlung ausgesprochen werden kann [10, 20].

#### Anterograde Irrigation

**Figure 1:**
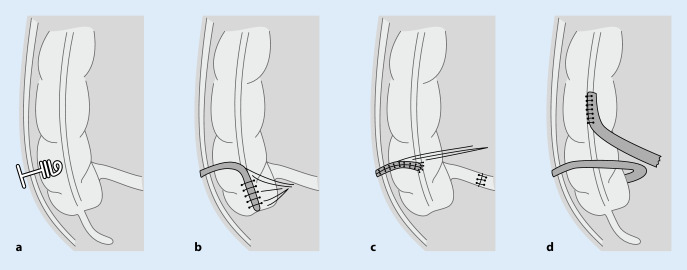

Ein Therapieansatz, ursprünglich für Kinder mit Spina bifida entwickelt, stellen anterograde Irrigationen („antergrade continent enema“, ACE) dar [25, 41, 49]. Entweder kann diese per endoskopisch eingebrachter perkutaner Zökostomie oder chirurgisch angelegter Stomata erfolgen [8, 20]. Die endoskopisch oder im Rendezvous-Verfahren implantierten Cope-loop-Katheter sind aus unserer Sicht als minimal-invasive, aber nicht langfristige Lösung bei nicht irrelevanter Rate an septischen Komplikationen (Abdominalwandinfektionen und Peritonitis) anzusehen und können nach erfolgreicher Testphase gegen einen sog. Chait-Katheter (Abb. 1a) gewechselt werden. Der Chait-Katheter kann aber auch unmittelbar operativ, vorzugsweise minimal-invasiv und auch im linksseitigen Kolon, eingebracht werden. Hier wurden bei Patienten mit NBD durchaus gute Erfolge beschrieben [38].

Eine Alternative zu den katheterbasierten ACE stellen unterschiedliche kontinente Stomata, wie z. B. das Malone-Appendikostoma (MACE) mit entsprechenden Modifikationen, zur Verfügung (Abb. 1b; [17, 33, 42]). Ein wesentliches Problem stellt die im Laufe der Zeit häufig entstehende narbige Enge dar, die ein Katheterisieren erschweren oder verhindern kann (5–50 %; [16, 20]). In einer Übersichtsarbeit von Paris und Kollegen wurde der Zeitaufwand der Abführmaßnahmen von 170–190 min auf 28–45 min reduziert. Die Kontinenzrate liegt bei 60–93 %, was zu Verbesserungen der Lebensqualität bei etwa 80 % der Patienten führte [20]. Bei etwa 30 % der Patienten musste eine operative Revision, ein Verschluss oder eine ableitende Stomaanlage im Verlauf erfolgen [20, 41].

Bei Patienten nach Appendektomie, zu kurzem Appendix oder adipösen Bauchdecken stehen noch andere Methoden zur Verfügung, wie das Monti-ACE, bei dem ein Ileumsegment umgeformt und als Irrigationsstoma ausgeleitet wird (Abb. 1c; [34, 43, 53]). Eine kürzlich beschriebene Variation stellt die Anlage einer endständigen distalen Ileostomie mit nachfolgender Ileoaszendostomie dar (Abb. 1d; [50]).

Diese Methoden werden hierzulande bisher wenig eingesetzt, die Evidenz für Erwachsene im Allgemeinen und insbesondere für Erwachsene mit Querschnittlähmung ist gering und basiert im Wesentlichen auf retrospektiven Fallserien [5, 42, 51]. In der einzigen, die katheterbasierten Varianten mit den Stomaanlagen vergleichenden Studie zeigten sich keine wesentlichen Unterschiede in Bezug auf Kontinenz- und Komplikationsraten [24].

#### Resezierende, „funktionelle“ Kolonchirurgie

Der Stellenwert resezierender Methoden im Sinne einer funktionellen Dickdarmchirurgie bei Patienten mit Querschnittsyndrom ist nur durch Fallserien belegt, die Evidenz also gering. Hierbei sind im Wesentlichen (erweiterte) linksseitige Kolektomien und Rektumresektionen in retrospektiven Serien beschrieben, allerdings wurde bei allen Patienten ein endständiges Stoma angelegt. Im Vergleich mit Daten aus der Literatur zeigte sich keine erhöhte Rate an Morbidität und Letalität [39, 56]. Relevante Daten zu Resektionen und Wiederherstellung der Kontinuität, z. B. Sigmaresektionen oder linksseitige Hemikolektomien als individualisierte Therapien bei entsprechenden morphologischen Veränderungen wie Elongation und Distensionen, liegen bis dato nicht vor. Die Gruppe um Negosanti empfiehlt grundsätzlich die Resektion der linken Flexur bis zum oberen Rektum und Anlage eines Transversostomas [39].

Die Evidenz für resezierende Eingriffe bei nichtquerschnittgelähmten Patienten ist nur wenig besser, es liegen nur retrospektive Studien und Fallserien vor [46]. Die meisten Erfahrungen liegen zu subtotalen Kolektomien mit ileorektaler Anastomose vor, sodass dies als Standardverfahren gilt. Die in der Normalpopulation schon mit 15 % angegebene postoperative Stuhlinkontinenz muss umso mehr bei den Querschnittgelähmten in der operativen Planung berücksichtigt werden [46]. Die Daten zur segmentalen, linksseitigen Kolonresektion bei chronischer Obstipation sind uneinheitlich und reichen von sehr schlechten bis guten Ergebnissen. Wenn eine linksseitige Kolonresektion erfolgen soll, sollte das Resektionsausmaß mittels Markerverteilung bei der radiologischen Kolontransitdarstellung bestimmt werden, wobei hier das Risiko der Dilatation des Restkolons besteht [46].

Wenn Eingriffe mit Herstellung einer Anastomose bei z. B. jungen Patienten mit explizitem Wunsch der Erhaltung der Kontinuität durchgeführt werden sollen, gilt insbesondere, eine gute und gründliche Diagnostik unter Berücksichtigung der operativen Morbidität durchzuführen, zumal mit der Anlage eines Kolostomas eine sichere und gut validierte Technik zur Verfügung steht.

#### Kolostomaanlage

Bei etwa 10 % aller querschnittgelähmten Patienten erfolgt die Anlage eines endständigen Kolostomas [40]. Wenn das Stoma lediglich passager z. B. zur Behandlung von Dekubitus angelegt wird, kann es auch doppelläufig sein, die möglichen Vor- und Nachteile sollten zuvor eng mit dem Patienten besprochen werden, zumal es nur bei einem geringen Teil der Patienten zu einer Rückverlagerung kommt [9]. Die Stomaanlage führt bei sorgfältiger Indikationsstellung unter Berücksichtigung der doch nicht unerheblichen Morbidität von bis zu 38 % aber zu einer signifikanten Steigerung der Lebensqualität bei Reduktion des Zeitaufwandes der abführenden Maßnahmen von etwa 110 min auf 12 min pro Tag, sodass aus unserer Sicht durchaus frühzeitig an diese Möglichkeit gedacht werden sollte [4, 13, 20, 44, 45, 55]. Auch die Zahl der subileusbedingten Krankenhausaufenthalte reduziert sich deutlich. Auf die Position des Stomas sollte in Hinsicht auf die zumeist liegende und sitzende Position der Patienten und deren Bewegungseinschränkung der oberen Extremität, unter Einbeziehung eines erfahrenen Stomatherapeuten, besonders geachtet werden [6]. Zur Verkürzung der Kolonpassage und aufgrund der zumeist im Oberbauch gelegenen günstigeren Positionierung, bevorzugen wir bei chronischen dilatativen Darmwandveränderungen des absteigenden Kolons die laparoskopische Anlage eines Transversostomas, auch wenn in der Literatur zumeist die Anlage eines Sigmoidostomas empfohlen wird [37, 45].

Bei der Stomaanlage sollte auch in Abhängigkeit von der Lähmungshöhe und demzufolge bestehenden Innervation der Bauchdecke oder Spasmen die prophylaktische Einlage eines Netzes erwogen werden, da bei den Patienten mit Bauchdeckenbelastung mindestens eine vergleichbar hohe Rate parastomaler Hernien zu erwarten ist [27].

Die Versorgung der häufigen Komplikationen wie Stomaprolaps und parastomale Hernierung unterscheiden sich nicht von denen nicht querschnittgelähmter Patienten, lediglich kann bei den Hernien bei fehlender Bauchdeckenbelastung eine operative Versorgung zurückhaltend indiziert werden.

### Das akute Abdomen bei Patienten mit Querschnittlähmung

Viszeralchirurgisch behandelbare akute Krankheitsbilder können in der Phase des spinalen Schocks, aber auch in der chronischen Phase auftreten und stellen, ob der querschnittspezifischen Charakteristika, besondere Anforderungen an die Diagnostik und Therapie der Patienten.

Es wird angenommen, dass es bezüglich der Häufigkeit der meisten viszeralchirurgischen Krankheitsbilder keinen relevanten Unterschied zur Normalbevölkerung gibt [36]. Wenn man sich vergegenwärtigt, dass das akute Abdomen im Allgemeinen definiert wird als akuter Bauchschmerz, Abwehrspannung und Kreislaufbeeinträchtigung, werden die Schwierigkeiten, eine akute abdominalchirurgische Erkrankung als solche zu erkennen, schnell klar. Insbesondere beim kompletten Querschnitt > Th(Thorakalsegement)7 muss beachtet werden, dass vom Patienten selbst keine Äußerung von Bauchschmerzen zu erwarten ist und die Diagnose häufig verzögert mit entsprechendem Einfluss auf die Mortalität erfolgt [1, 52].

Üblicherweise kommt es zu einem unklaren septischen Krankheitsbild mit allgemeinem Unwohlsein, einem aufgetrieben Abdomen mit Störung der Atemmechanik, Abführproblemen, Übelkeit und Erbrechen im Sinne eines Ileusbildes und natürlich auch Fieber und Tachykardie [47]. Symptom der chronischen Obstipation können paradoxe Diarrhöen ebenso wie Stuhlverhalt sein. Eine signifikante Änderung der auftretenden Spastik kann auch einen Hinweis auf eine intraabdominelle Pathologie geben [36]. Einige Autoren beschreiben auch das Auftreten einer autonomen Dysreflexie als mögliches frühes Zeichen einer akuten abdominellen Affektion [28].

Nicht selten und insbesondere bei hohem Fieber sind die bei diesen Patienten häufig auftretenden Harnwegsinfektionen die Ursache für derartige paralytischen Ileusbilder, aber auch andere septische Ursachen, wie Pneumonie oder ausgedehntem Dekubitus, können reaktiv Darmtransportstörungen induzieren. Allerdings schließt ein pathologischer Urinbefund eine intraabdominelle Affektion auch nicht aus [36].

Die abdominelle Untersuchung kann aufgrund von Spastiken in der Bauchdeckenmuskulatur oder auch Atonie unter Umständen nicht zielführend sein.

Begonnen wird, wenn möglich, mit der Anamnese. Gegebenenfalls muss eine Fremdanamnese erhoben werden. Gefolgt wird dies von der ausführlichen körperlichen Untersuchung unter Berücksichtigung der möglichen fehlenden Sensibilität. Nicht zu vergessen ist die digitale rektale Untersuchung, insbesondere wenn die anorektale Funktion nicht bekannt ist. Die weitere Diagnostik beinhaltetet die Labordiagnostik einschließlich der laborchemischen Entzündungszeichen (Leukozyten, C‑reaktives Protein und Prokalzitonin), denen aufgrund der unspezifischen Klinik eine wesentliche diagnostische Bedeutung zukommt [3].

Bei der Sonographie ist darauf hinzuweisen, dass aufgrund des häufigen Meteorismus eine geringere Aussagekraft besteht, sodass frühzeitig die CT-Diagnostik in Betracht gezogen werden muss. Trotz stetiger Verbesserung der diagnostischen Genauigkeit der bildgebenden Verfahren sollte im Zweifel die Indikation zur explorativen Laparoskopie gestellt werden [23].

Erwähnenswert ist die erhöhte Inzidenz für die Bildung von Gallenblasen-Sludge in den ersten 6 Monaten nach Schädigung des Rückenmarks. Dies korreliert nicht mit Dauer oder Höhe der vorliegenden Schädigung und auch nicht mit anderen sonst prädisponierenden Faktoren wie Alter, Diabetes und Adipositas, wohl aber mit dem Schweregrad der Verletzung [47].

Kommt es zu einer akuten Komplikation der chronischen Obstipation, wie Megakolon, Volvolus und Dünndarmileus bei Koprostase im Colon ascendens, sollten in Abhängigkeit des zuvor bestehenden Beschwerdebildes Resektionen des chronisch veränderten Darmes erfolgen. Bei der Abwägung, ob ein endständiges Stoma oder eine Anastomosierung mit oder ohne protektives Stoma erfolgen kann, sollte immer die Grundmorbidität der Patienten berücksichtigt werden. Wir verfahren hier deutlich vorsichtiger (im Sinne einer Vermeidung von Anastomosen), als man dies vielleicht in der Normalbevölkerung tun würde.

### Bariatrische Chirurgie

Die Auswirkungen auch vermeintlich geringer Gewichtszunahmen auf Mobilität und Lebensqualität bei Patienten mit einem Querschnittsyndrom sind weitaus gravierender als bei der „laufenden“ Bevölkerung [32]. Schnell reicht die Kraft der Arme der Patienten, aber auch der Pflegenden nicht mehr aus für den Transfer in den Rollstuhl, die kostenintensiven Hilfsmittel werden zu klein oder erst erforderlich, wie Transfergerätschaften. Die Möglichkeiten der Gewichtsabnahme durch Ernährungstherapie, Verhaltensumstellung und Bewegung sind durch die Querschnittlähmung noch eingeschränkter, sodass adipositaschirurgische Operationen ein effektives und sicheres Mittel darstellen, den Patienten zu helfen [48].

Die Diagnosekriterien der Leitlinien müssen hier angepasst werden, um dem speziellen Patientengut gerecht zu werden [11]. So kann z. B. der Body-Mass-Index nur sehr eingeschränkt angewendet werden, die Atrophie der Muskulatur verzerrt den Körperfettanteil deutlich [32].

Die Fallserien bariatrischer Operationen sind überschaubar, sodass man hier nur von einem sehr begrenzten Evidenzniveau sprechen kann [48]. Die zukünftig anzunehmenden steigenden Fallzahlen müssen gut in Studien und Registern dokumentiert und begleitet werden, um den Nutzen besser beurteilen zu können. Wir präferieren in unserem Patientengut die Durchführung einer laparoskopischen Magenschlauchbildung, um den Eingriff in die pathophysiologischen Besonderheiten der Patienten so gering wie möglich zu halten. Es finden sich in der aus kleinen Einzelfallbeschreibungen und Fallserien bestehenden Literatur aber ebenso Roux-Y-Magenbypass-Operationen und „duodenal switches“ [48].

### Perioperative Besonderheiten

Findet sich eine chirurgisch zu therapierende Indikation, müssen perioperativ einige Besonderheiten bei querschnittgelähmten Patienten beachtet werden. Es kommt nicht selten zum Blutdruckabfall bei Narkoseeinleitung und postoperativ zu schmerz- oder spastikbedingtem Blutdruckanstieg. Außerdem können bestehende Deformitäten oder Kontrakturen sowohl die Narkose als auch die Operation selbst durch eingeschränkte Lagerbarkeit (Steinschnittlage oder Einschränkungen des Bewegungsausmaßes bei Laparoskopien) beeinträchtigen.

Die häufig fehlende Klinik bei einer Peritonitis erfordert aus unserer Sicht auch eine großzügigere Einlage von Zieldrainagen, als die allgemeine Evidenz dies empfehlen würde.

Eine Besonderheit der Patienten stellt das prolongiert auftretende postoperative paralytische Ileusbild dar, das sich in aller Regel nach Ausschluss ursächlicher postoperativer Komplikationen gut konservativ mittels medikamentöser Stimulation und nasogastraler Entlastung behandeln lässt.

Zudem handelt es sich besonders bei Langzeitquerschnittgelähmten häufig um chronisch Kranke mit weiteren Risikofaktoren. Es gibt ein deutlich erhöhtes Risiko für postoperative Wundheilungsstörungen und Wundinfekte, da bei lähmungsbedingter Störung der Trophik zusätzlich chronische septische Foci vorliegen können wie z. B. rezidivierende urogenitale Infekte, Bronchitiden und Dekubitus [2, 56]. Daher hat bei den Patienten trotz des (vermeintlichen) Wegfalls der schmerzbedingten postoperativen Einschränkungen durch eine Laparotomie das minimal-invasive Vorgehen eine besondere Bedeutung und sollte, wenn möglich, angewandt werden.

## Schlussbetrachtung

Querschnittgelähmte Patienten können nicht mit nichtgelähmten Patienten gleichgesetzt werden. Es handelt sich um ein besonderes Patientenkollektiv, welchem sowohl in der Anfangsphase der Erkrankung besondere Aufmerksamkeit hinsichtlich abdomineller Komplikationen geschenkt werden muss als auch im weiteren Verlauf bezüglich der NBD und seiner Folgen. Die Therapie der NBD ist zunächst eine Domäne der konservativen Medizin, nach deren Ausschöpfen stehen aber verschiedene chirurgische Therapien zur Verfügung. Bis auf die Anlage von Kolostomata ist die Evidenz für die Interventionen und operativen Behandlungen gering. Es herrscht folglich ein dringender Bedarf an der Durchführung hochwertiger Studien und dem Aufbau nationaler Datenbanken, um die Behandlung der therapierefraktären NBD mit ihren massiven Einschränkungen der Lebensqualität zu verbessern.

Alle abdominalchirurgischen Krankheitsbilder können bei querschnittgelähmten Patienten genauso vorkommen wie bei nichtgelähmten. Die Diagnostik insbesondere im Hinblick auf die körperliche Untersuchung ist jedoch erschwert und häufig verzögert, was zur erhöhten Morbidität und Mortalität der einzelnen Erkrankungen führen kann. Frühzeitig sollten daher in der Diagnostik der Erkrankung, aber auch im Rahmen der postoperativen Komplikationsdetektion bildgebende Verfahren eingesetzt werden.

Die stationäre Versorgung der querschnittgelähmten Patienten ist komplex und auch durch die häufig bestehenden Berührungsängste durch das zumeist im Umgang ungeübte viszeralchirurgische Personal kann es dazu kommen, dass man den Patienten nicht in ausreichendem Maße gerecht werden kann. Hier stehen Querschnittgelähmtenzentren mit entsprechender interdisziplinärer Expertise zur Verfügung, in die nach initialer akutmedizinischer Versorgung eine Verlegung erwogen werden kann, oder die im Falle elektiver Eingriffe für eine Beratung und Behandlung hinsichtlich viszeralchirurgischer Fragestellungen zur Verfügung stehen.
