# Erratum zu: Der querschnittgelähmte Patient. Besonderheiten der viszeralchirurgischen Diagnostik und Therapie

**DOI:** 10.1007/s00104-021-01392-y

**Published:** 2021-04-01

**Authors:** Julia Seifert, Ralf Böthig, Stefan Wolter, Jakob R. Izbicki, Roland Thietje, Michael Tachezy

**Affiliations:** 1 Abteilung für Allgemein und Viszeralchirurgie, BG Klinikum Hamburg, Hamburg, Deutschland; 2 Klinik für Allgemein‑, Viszeral- und Thoraxchirurgie, Universitätsklinikum Hamburg-Eppendorf, Hamburg, Deutschland; 3 Abteilung für Neuro-Urologie des Querschnittgelähmten-Zentrum, BG Klinikum Hamburg, Hamburg, Deutschland; 4 Querschnittgelähmten-Zentrum, BG Klinikum Hamburg, Hamburg, Deutschland

**Erratum zu:**

**Chirurg 2021**

10.1007/s00104-021-01364-2

Der Name des Erstautors Ralf Böthig ist im ursprünglich publizierten Beitrag nicht korrekt genannt worden. Die Autoren bitten den Fehler zu entschuldigen.

